# Validity and reliability of the Output sport device for assessing drop jump performance

**DOI:** 10.3389/fbioe.2022.1015526

**Published:** 2022-10-17

**Authors:** Raynier Montoro-Bombú, Adam Field, Amândio Cúpido Santos, Luis Rama

**Affiliations:** ^1^ Faculty of Sport Sciences and Physical Education, University of Coimbra, Coimbra, Portugal; ^2^ Division of Sport, Exercise and Nutrition Sciences, School of Human and Health Sciences, University of Huddersfield, Huddersfield, United Kingdom; ^3^ Research Unit for Sport and Physical Activity (CIDAF), Coimbra, Portugal

**Keywords:** accelerometer, jump height, ground contact time, flight time calculation, reactive strength index, inertial measurement unit, plyometric exercise

## Abstract

The devices for measuring plyometric exercise in field conditions are becoming increasingly prevalent in applied research and practice. However, before the use of a device in an applied setting, the validity and reliability of such an instrument must be determined. The study aimed to assess the validity and reliability of the Output Sport, an inertial measurement unit (IMU), through comparisons with a force plate for research purposes. A repeated measure test-retest study was performed. Reliability was assessed during single-session trials (i.e., intrasession reliability). A total of 34 national/university level athletes (13 females, 21 males) performed three drop jumps with a fall from 30 cm while both devices recorded ground contact time (GCT), flight time (FT), jump height (HJ), and reactive strength index (RSI). T-tests demonstrated that data collected from the IMU device were significantly different to the force platform for all reported variables (all *p* < 0.01). The intraclass correlation coefficients (ICC) demonstrated good-to-excellent reliability, but with a large range of confidence intervals (CI 95%) for GCT (0.825, 0.291–0.930), FT (0.928, 0.756–0.958), HJ (0.921, 0.773–0.964), and RSI (0.772, 0.151–0.907). The Bland-Altman test showed that the device overestimated contact times and underestimated the other variables. Upon landing, greater ground contact times (i.e., ≥0.355ms) were associated with higher reliability. These results suggest that a single IMU can be used to track changes somewhat accurately and reliably in jump metrics, especially when the GCT is greater than 0.355ms. It is recommended that before practitioners and trainers use the device as a cost-effective solution in the field, further research should be carried out to evaluate a range of data on the type of exercise to be performed.

## Introduction

Measuring vertical jump performance accurately in field conditions, particularly the drop jump (DJ), is increasing in interest among coaches and practitioners of high-level sports. Vertical jump performance is one of the most studied measures within the plyometric literature (Allen et al., 2012; Gillen et al., 2019; Frayne et al., 2021; Montalvo et al., 2021). A possible explanation for this increased interest could reside in the practical applicability of the measure and its potential to differentiate real-time magnitudes of vertical impulse and ground contact time (Wallace et al., 2010), jump height performance (Bosco et al., 1983; Bobbert et al., 1987; Linthorne, 2001; Glatthorn et al., 2011; Gu et al., 2021), reactive strength index (RSI) (Ebben and Petushek, 2010; Ball and Zanetti, 2012; Byrne et al., 2017; Montalvo et al., 2021), provide an assessment of vertical and/or leg stiffness, (McMahon and Cheng, 1990; Arampatzis et al., 2001; Hobara et al., 2010; Lloyd et al., 2011), intramuscular coordination (Markovic and Mikulic, 2010) and neuromuscular fatigue (Horita et al., 2003; Wang et al., 2021).

For the analysis of vertical jump performance, force plates (FP) are traditionally used and are considered the gold standard (Arampatzis et al., 2001; Busko et al., 2010; Hobara et al., 2010). Such instruments can report—with a high level of accuracy—force production, ground contact time (GCT) and, through mathematical derivations, can estimate the jump height (HJ), acceleration, load quantification, velocity and centre of mass (Jorgensen et al., 2021).

Previously validated systems that provide reliable information (Pueo et al., 2020) and are based on flight time (FT) calculations (Bosco et al., 1983; Linthorne, 2001; Pueo et al., 2020) have been used and have demonstrated practicality (Allen et al., 2012; Gillen et al., 2019; Frayne et al., 2021; Montalvo et al., 2021). With the advancements in science and technology, mobile applications (Haynes et al., 2019) and inertial measurement units with integrated accelerometers, gyroscopes and magnetometers are being used to measure jump analyses (Choukou et al., 2014; O'Reilly et al., 2017a; O'Reilly et al., 2017b; O'Reilly et al., 2018; Johnston et al., 2019). Studies have demonstrated the validity and reliability of different accelerometer devices for velocity-based training (Balsalobre-Fernandez et al., 2016; Hughes et al., 2019; Lake et al., 2019), injury detection (Li et al., 2016), and monitoring sleep quality (Reimers et al., 2021) with high accuracy. Additionally, separate investigations have involved the validation of the functionality of these devices for the measurement of jump metrics (Choukou et al., 2014; Lake et al., 2018; Montalvo et al., 2021; Montoro-Bombú et al., 2022). A new commercially available inertial measurement unit (IMU), namely the Output Sport measurement device, has been used for fatigue assessment (Buckley et al., 2017), injury prevention (Whelan et al., 2016), agility (Johnston et al., 2019), velocity-based training (O'Reilly et al., 2015), lower limb exercise assessments (O'Reilly et al., 2017a; O'Reilly et al., 2017b; O'Reilly et al., 2018), and postural control and balance evaluations (Johnston et al., 2019). However, to date, there are no reports of the validation for measuring a single RSI effort (i.e., aside from a 10–5 RSI assessment), FT and GCT for the IMU measurement device.

If the concurrent validity is confirmed, the device could be a valuable tool for coaches who cannot directly (i.e., face-to-face) engage with their athletes for several reasons (e.g., international coaches and practitioners, COVID-related isolations etc.). Such remote engagement can be achieved due to the device’s’ web-integrated data monitoring system and the subsequent detailed information able to be shared digitally between athletes and practitioners. Furthermore, the device presents an easily transportable alternative to some of the other larger and less manageable jump measure technologies on the market. This device could be of enormous interest accounting for the intense travel schedules of elite sports (Montoro-Bombú et al., 2022), providing an opportunity for monitoring athletes while away from their usual training facilities.

The study aimed to assess the validity and reliability of the Output Sport during a drop jump. This study represents an initial crucial step toward using the Output Sport measurement device before its integration within the training environment.

## Materials and methods

### The experimental approach to the problem

The study followed the same procedures outlined in previous research related to the validation and intra-session reliability of jumps assessment equipment (Montoro-Bombú et al., 2022). All participants performed three DJ (for RSI assessment), with jump parameters recorded simultaneously with an FP and the IMU measurement device. The same evaluator conducted all experimental evaluations.

### Subjects

Thirty-four national and university-level athletes, 13 females and 21 males (age: 22 ± 4 years, stature: 1.77 ± 0.07 m, mass: 72.3 ± 7.7 kg, BMI: 22.9 ± 2.4) with one or more years of training experience were recruited for participation. Table 1 presents the characteristics of the sample. To be included in the study, athletes were required to have no contraindications to exercise, such as not suffering from lower-extremity injuries in the preceding 6 months. Participants were also instructed to avoid intense exercise 24 h before the assessment session. All participants were fully informed and familiarized with the experimental procedures prior to participation before providing written consent. The research was conducted following the ethical principles of the Declaration of Helsinki (2010). It was approved by the Faculty of Sport Sciences and Physical Education at the University of Coimbra ethics committee (code: CE/FCDEF-UC/00802021).

**TABLE 1:** Anthropometric characteristics of the subjects.

	Women (*n* = 13)	Men (*n* = 21)	Total (*n* = 34)
Age (yrs.)	21 ± 4	22 ± 4	22 ± 4
Mass (kg.)	65.8 ± 6.5	76.4 ± 5.1	72.3 ± 7.7
Stature (m.)	1.71 ± 0.04	1.81 ± 0.05	1.77 ± 0.06
BMI (kg.m^−2^)	22.3 ± 2.4	23.3 ± 2.3	22.4 ± 2.4

### Procedures

Before experimentation, participant stature, body mass and age were collected. Stature was measured using a stadiometer with an accuracy of 0.1 cm (Bodymeter 206, SECA, Hamburg, Germany). Body mass was assessed using a SECA scale (Hamburg, Germany), and body mass index was calculated according to previous protocols (Salami et al., 2010). After a comprehensive explanation, a standardized warm-up was undertaken, which consisted of 5 min low intensity running, followed by a 10 min dynamic stretching sequence (Turki et al., 2011). Subsequent jump analyses were recorded. Each trial comprised three drop jump efforts performed by each athlete. Each jump was interspersed with 2 min of passive recovery.

**FIGURE 1:**
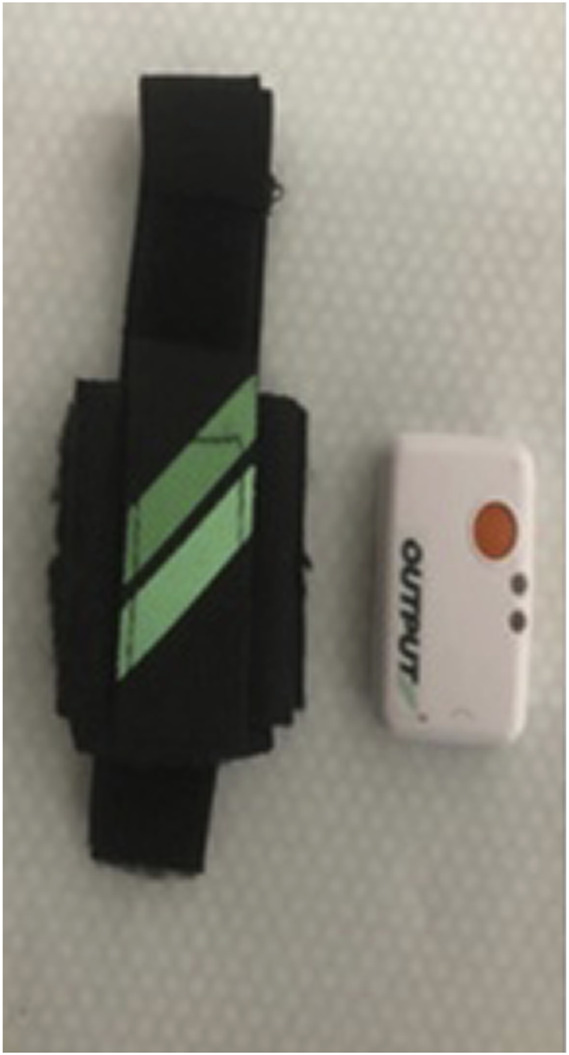
Separation of the mobile inertial unit OUTPUT at 10 cm from the force plate.

**FIGURE 2:**
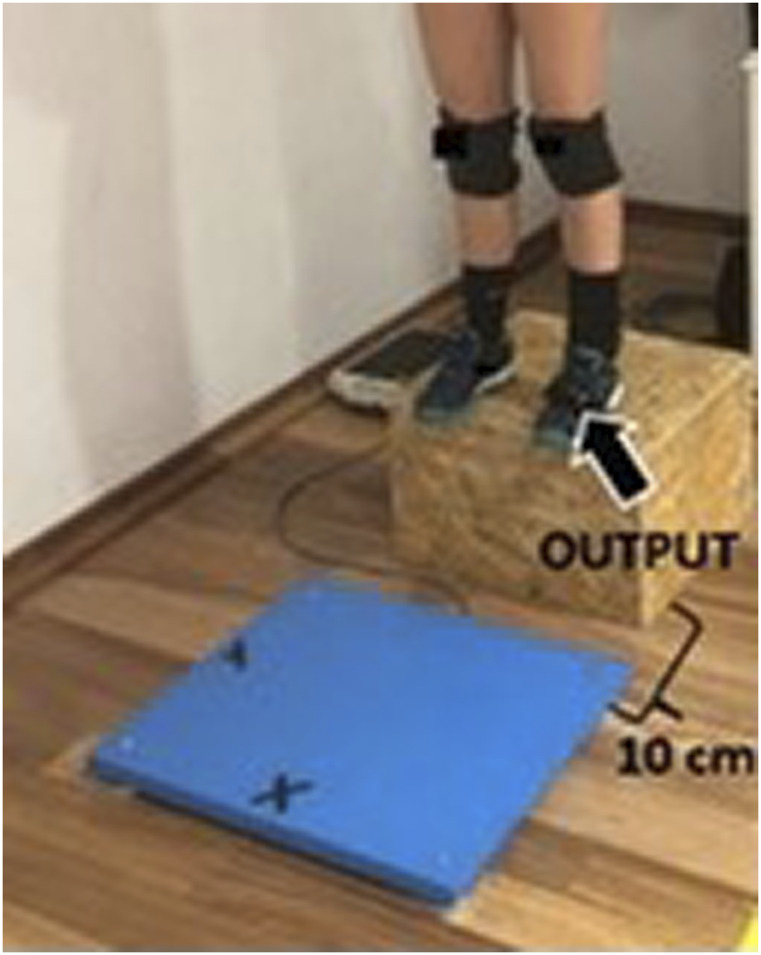
Placement of the OUTPUT mobile inertial unit on the left leg.

**FIGURE 3:**
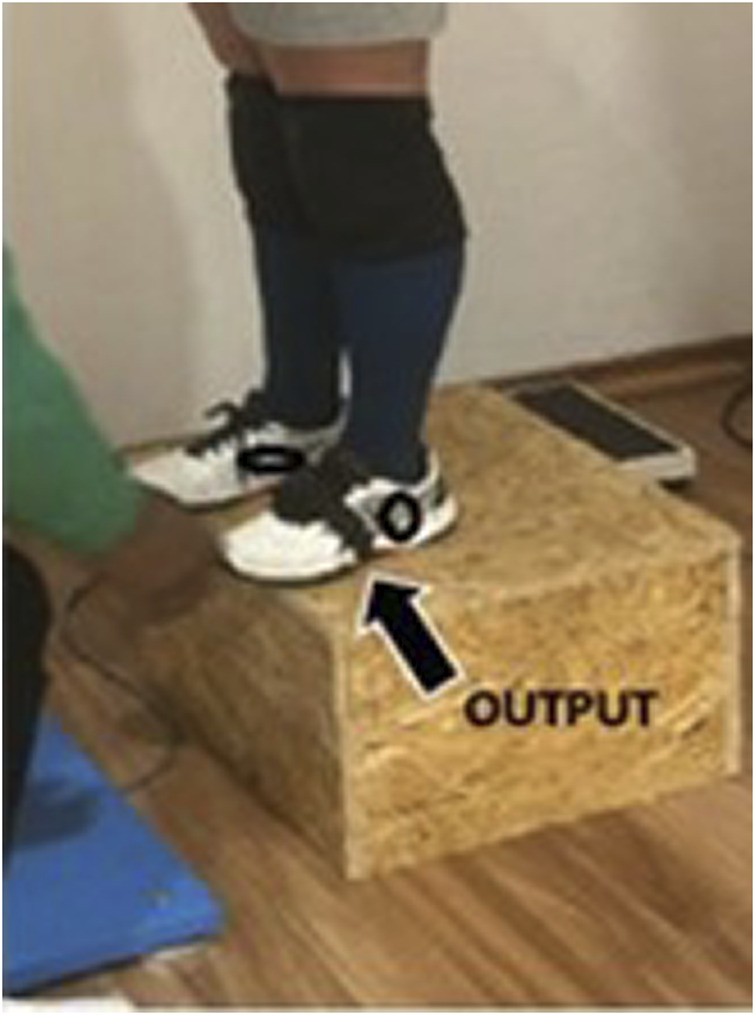
OUTPUT mobile inertial unit and the positioning strap.

Each athlete performed three jump attempts with both devices synchronized. The FP was placed on a flat, compact surface level placed 10 cm adjacent to the side of the box for the DJ. (Figures 1–3). The IMU measurement device was carefully placed on the front left shoe (according to the manufacturer’s instructions) of each participant preventing the effect of movement during each attempt. Before each jump attempt, the device was manually readjusted as per the manufacturer’s’ recommendations. The synchronization strategy allowed simultaneous data recordings for each jump attempt on both the FP and the IMU measurement device.

Following established standards, a 30 cm high platform was used for each DJ trial (Jarvis et al., 2016; Ramirez-Campillo et al., 2018). The DJ performed with the hands placed at the waist to avoid the influence of the arm swing on HJ. For consistency in the drop jump protocol, the first leg that initiate the fall was strictly the right leg; the attempt was invalidated if the left leg started the fall, or if the landing occurred visually with a single leg. The RSI was calculated from the DJ with rebound, with participants asked to maximize HJ while minimizing GCT.

### Instruments

Data were collected using an FP (Kisler Instrument AG, Winterthur, Switzerland) and displayed in real-time at a sampling rate of 1,000 Hz using an interface box (Kistler,Model 9260AA6, Winterthur, Switzerland). Data were analyzed using Bioware 5.3.2.9 software following the manufacturer’s’ instructions. The IMU measurement device sensor (sensor dimensions: 51 × 34 × 14 mm, mass: 24 g) was used in conjunction with the FP to record jump efforts. The IMU device contained two integrated accelerometers (one ± 2 G, 16 bit and the other ± 16 G, 14 bit), a gyroscope (± 250–2000 DPS, 16-bit) and a magnetometer (± 49 GAUSS, 16-bit, sampling rate: 50–1,000 Hz). An Android 7.0 device displayed the data and was exported as a. *csv* file for posterior analyses.

The IMU device calculated HJ using the following Eqn. 1:

$$Jump\;height=9.81/8\times {FLIGHT\;TIME}^{2}$$ (1)

The RSI is calculated using Eqn. 2 expressed in meters per second [16]:

RSI=HJ(cm)/GCT(ms) (2)

### Statistical analysis

Data are presented as mean ± SD and (if indicated) SE. The Shapiro-Wilk test was used to ensure a normal distribution of the data. The statistical power was performed with the statistical software G*Power version 3.1.9.7. The *a priori* statistical power was based on the difference between two dependent means (pairwise). A beta value of 85% was achieved, with an alpha of 0.05 and a moderate effect size (0.6) and demanding a sample size of 27 participants. The calculation for inter-device validity was determined using the following strategy: 1) the mean differences through the T-test, 2) the Bland-Altman plot, and 3) the intraclass correlation coefficient (ICC) calculation. The one-sample t-test was used to check whether the measurements reported by the devices were statistically ≠ 0, as a first indicator of validity. A Bland-Altman plot was used to check the level of between-device agreement. A simple linear regression model was run to check for potential biases between the two devices. The dependent variable was the mean differences, and the independent variable was the mean of the two measures. The significance level was established (*p* ≤ 0.05). The ICC was used in the analysis of all metrics (GCT, TF, HJ, and RSI). Sustained in the tercile calculation of the CGT data, we considered three groups according to CGT performance level and performed separate reliability analyses accordingly. The ICC estimates and their 95% confidence intervals based on the 2-way mixed-effect model of Shrout and Fleiss (1979). Thresholds for ICC were considered poor (<0.5), moderate (0.5–0.75), good (0.75–0.9) and excellent (<0.9). The strength of the correlations was interpreted as follows: r 0.00–0.10 was considered trivial, r 0.11–0.30 was considered small, r 0.31–0.50 was considered moderate, r 0.51–0.70 was considered large, r0.71–0.90 was considered very large, and r0.91–1.0 was considered nearly perfect Hopkins (2010). The standard error of the mean (SEM) was used to report the standard error of the measurements, together with the coefficient of variation (CV) that allowed to check the acceptable absolute reliability following previous recommendations (CV >10% = poor, 5–10% = moderate, <5% = good) (Banyard et al., 2017; Lake et al., 2019). The absolute agreement was chosen for the test-retest study design (Koo and Li, 2016). Alpha was set at *p* ≤ 0.05. Data analysis was performed with the statistical program SPSS, V.27.0 and the graphs were produced with the statistical software GraphPad Prism Version.9.4.0.

## Results

Table 2 shows the means ± SD of the variables registered by both devices. The means differences (MD) of measures (GCT, FT, HJ, and RSI) obtained by the IMU device and the PF were statistically different from zero (*p* ≤ 0.05), showing disagreement. This result could be considered the first step in demonstrating the lack of validity of IMU.

**TABLE 2:** Means ± SD of the kinematic variables registered by both devices.

Device	FT	GCT	HJ	RSI
OUTPUT	0.476 ± 0.062	0.333 ± 0.070	0.282 ± 0.072	0.878 ± 0.283
FP	0.496 ± 0.066	0.292 ± 0.070	0.307 ± 0.079	1.126 ± 0.436

**FIGURE 4:**
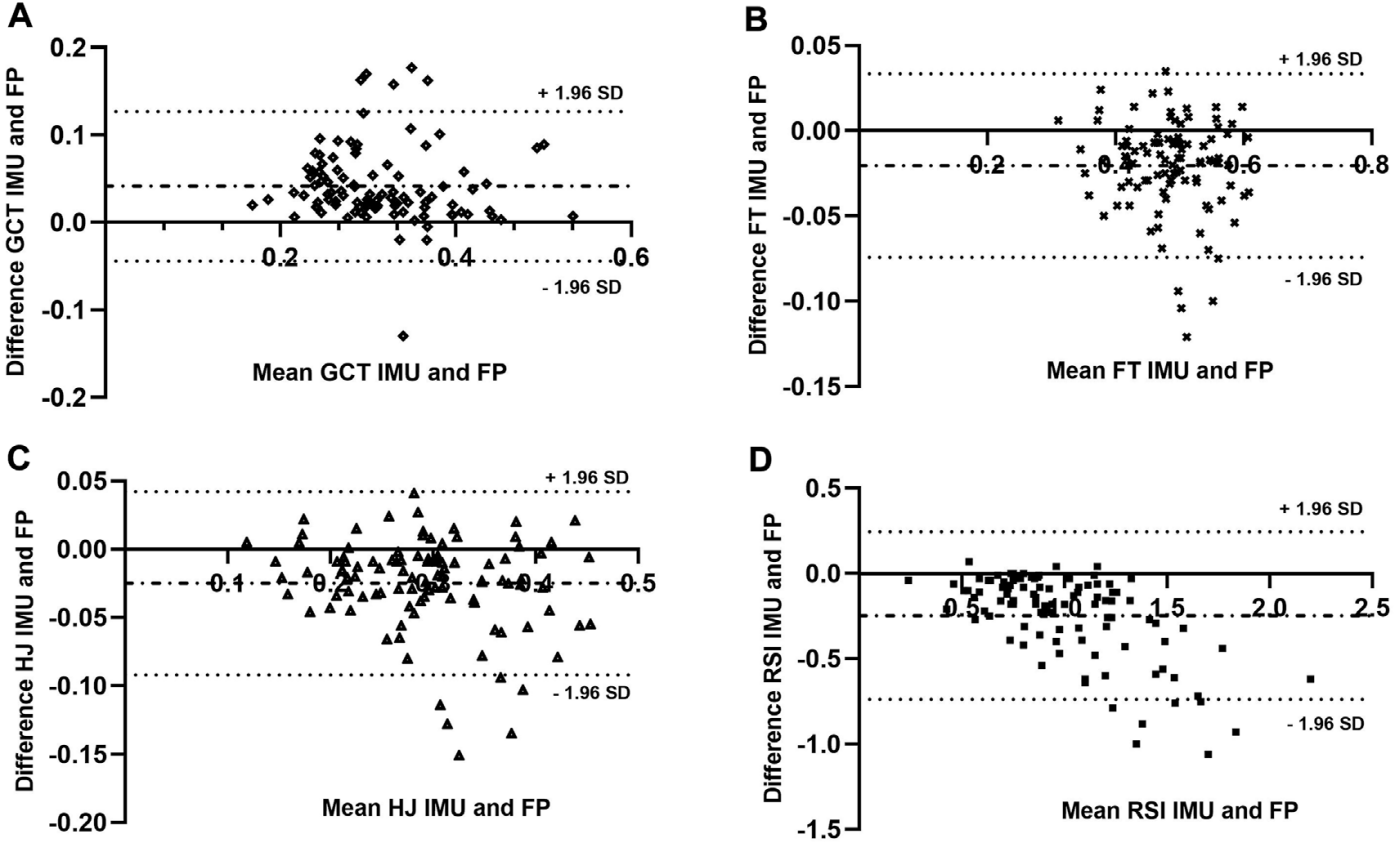
Bland Altman test. Measurements are obtained from the plate force and the OUTPUT sport. Representation **(A)** difference of Ground Contact Times (GCT) v/s mean GCT. Representation **(B)** difference of Flight Time (FT) v/s mean FT. Representation **(C)** difference of Jump Height (HJ) v/s mean of HJ. Representation **(D)** difference of Strength Reaction Index (SRI) v/s mean of RSI.

The Bland Altman (BA) analysis between the IMU and FP showed that 96.4% of the data were found to be within the limits of agreement (LOA) (Figure 4), with slight overestimation of GCT (Figure 1) and underestimation of FT and HJ (Figures 4B,C). The RSI showed a marked underestimation of the values compared to the force plate (Figure 4D). The LOA compromises the validity of the IMU measurements. Where GCT (MD = 0.041 m s; SD = 0.044 m s LB = 0.127 m s -UL = −0.044 m s); FT (MD = −0.020 m s; SD = 0.027 m s; LB = 0.033 m s–UL = -0.074 m s); HJ (MD = −0.025 m; SD = 0.034 m; LB = 0.0421 m–UL = −0.092 m), and RSI (MD = 0.246 m/s; SD = 0.251 m/s; LB = 0.245 m/s–UB =–0.739 m/s).

**FIGURE 5:**
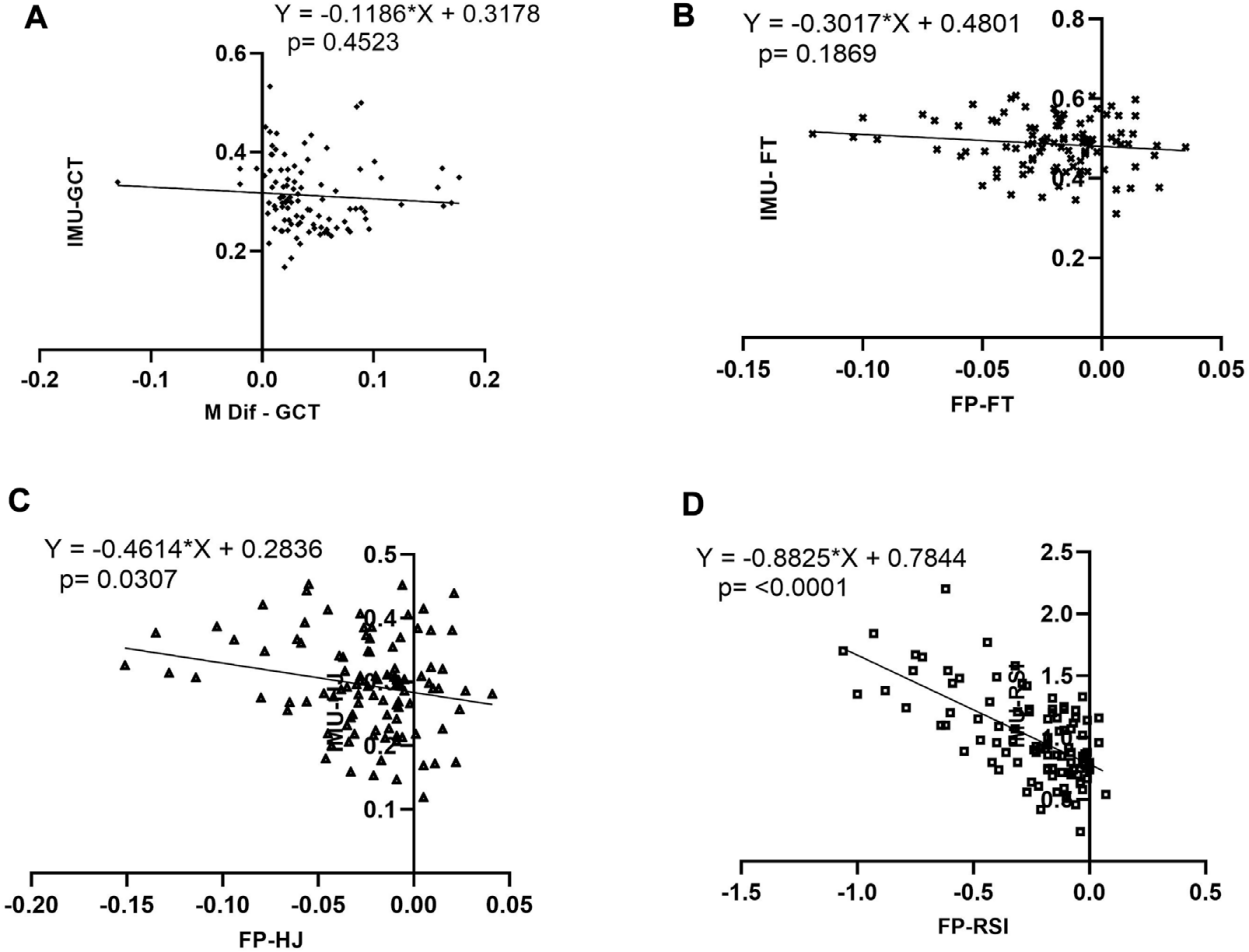
Simple linear regression (LR). The measurements are obtained from the difference of means vs. means for each. The **(A)** RL representation of GCT between IMU vs. FP; the **(B)** RL representation of FT between IMU vs. FP; the **(C)** RL representation of HJ between IMU vs. FP and the **(D)** RL representation of RSI between IMU vs. FP.

The potential bias between IMU versus PT measurements was analyzed using the simple linear regression model (Figure 5). It was found that GCT and FT did not present a bias of proportion GCT (*p* = 0.452; SEM = 0.063), FT (*p* = 0.186; SEM = 0.043), in opposite the HJ and RSI presented a bias of proportion HJ (*p* = 0.030; SEM = 0.045) and RSI (*p* = 0.0001; SEM = 0.056). Finally, good inter-measure reliability was found (Table 3). GCT (ICC = 0.825; CI = 0.291–0.93; IMU: [CV = 21.11%; SEM = 0.006] and FP [CV = 25.17%; SEM = 0.007]). The FT (ICC = 0.928; CI = 0.756–0.968; IMU: [CV = 13.16%; SEM = 0.006] and FP [CV = 25.17%; SEM = 0.007]). The HJ (ICC = 0.921; CI = 0.743–0.964; IMU: [CV = 25.56%; SEM = 0.007] and FP [CV = 25.80%; SEM = 0.007]). The RSI (ICC = 0.772; CI = 0.151–0.907; IMU: [CV = 32.22% SEM = 0.028] and FP [CV = 38.78%; SEM = 0.043]). The tercile calculation of the CGT rank data pointed to the cut values as follows: 1) GCT<0.297 m s; 2) CGT between 0.297 and 0.355 m s) and 3) GCT >0.355 m s (Table 3).

**TABLE 3:** Intraclass correlation coefficients and 95% confidence intervals for measures reported by the Output Sport device.

Contact time	≤0.297m s	0.297 and 0.355m s	≥0.355m s	General
Measures	ICC (IC 95%)	ICC (IC 95%)	ICC (IC 95%)	ICC (IC 95%)
GCT	0.442 (-0.145–0.730)	0.363 (-0.210–0.688)	0.585 (-0.076–0.824)	0.825 (0.291–0.930)
FT	0.841 (-0.085–0.954)	0.966 (0.786–0.989)	0.992 (0.984–0.996)	0.928 (0.756–0.958)
HJ	0.829 (-0.079–0.949)	0.966 (0.787–0.989)	0.992 (0.984–0.996)	0.921 (0.773–0.964)
RSI	0.591 (-0.230–0.855)	0.840 (-0.135–0.957)	0.763 (0.319–0.901)	0.772 (0.151–0.907)

GCT, ground contact time; FT , flight time; HJ , jump height; RSI, reactive strength index.

## Discussion

This study aimed to test the concurrent validity and reliability of the Output Sport measurement device against a traditional research-grade force platform approach. To the authors knowledge, this is the first validation study of this device for ground contact time, flight time, jump height and RSI during a drop jump protocol. Given the multitude of sporting devices on the market measuring aspects of performance, it is critical to assess the validity and reliability of such tools before transferring these instruments into practice. It is also crucial that researchers quantify the measurement error to ensure inferences can be interpreted within the confines of the equipment used. The findings demonstrate that although the IMU is somewhat valid and reliable, further research would need to be carried out before the Output Sport device can be incorporated into applied RSI research.

T-tests revealed significant differences for all measures between the Output Sport measurement device and the force platform. However, the Bland Altman analyses demonstrated that 96.66% of the recorded attempts are within the 95% LOA (Figure 1A). This indicates that the data may be reliable, but it is also recommended that the magnitude of the LOA should be considered alongside the mean values. LOA have not been reported in previous research (Balsalobre-Fernandez et al., 2016; Orser et al., 2020), therefore, these studies did not account for the variation in the estimate. In this investigation for each drop jump attempt performed with the Output Sport device, the IMU tended to overestimate GCT in relation to FP. This relates to the possibility of expecting high variations in the metrics according to the LOA reported here (0.127m s above mean values), which despite being within the confidence interval, might not have practical significance for high-performance sporting activity. Figures 4B–D denote a different trend with an underestimation observed for FT, HJ, and RSI. The limits are also far from the mean of the differences, which could indicate that the device can show an RSI with values of 0.24 above and 0.74 below the mean.

To investigate the possible nuances involved in the validity and reliability of the Output Sport device, analyses were individualised using the ICC index. ICC analyses have been commonly reported for validation studies (Lake et al., 2018; Pueo et al., 2020) as it reflects not only the degree of correlation but also the agreement between measurements (Koo and Li, 2016). The overall individualised ICC values are presented in Table 3.

The ICC demonstrated moderate-to-excellent reliability for most of the variable analysed, with moderate reliability observed for GCT. This estimate, according to previous studies, shows a good level of reliability (Shrout and Fleiss, 1979; Koo and Li, 2016). An important consideration is the confidence intervals, and it is recommended that studies observe this value before reliability is concluded. In the present study, we report ICC ranging from poor to excellent reliability, which indicates high variability. A closer examination of the results (Table 3) showed that the ICC tended to improve for FT and HJ as a function of increasing contact times (≥0.355 m s) as corroborated by flight time with GCT ≥0.355 m s.

It was found that the UMI device tends to improve data reliability with increasing GCT times, although it may not be helpful for practitioners intending to use the device to assess reactive jumping metrics. However, reliable, and valid assessments can be made for populations interested in jump height to evaluate changes in falls strictly with height-focused jumps rather than the reactive component as the primary target. However, given conflicting results in the literature and the present investigation, further research supporting or opposing the use of the Output Sports device is required.

These data suggest that the Output Sport device may represent acceptable validity, demonstrating a GCT above 0.355m s during drop jumps or plyometric exercise. This is an essential practical finding, especially when considering the possibility of using these exercises for jumps involving high altitudes where contact time is longer or for jumps with a drop not seeking a reactive component as their primary objective.

## Conclusions

The results of this study demonstrate that the Output Sport device could be used in practice to provide valid and reliable jump measurements, but further work may be needed to verify the current findings. This instrument could be implemented within the applied setting as a cost-effective and valid tool for assessing plyometric capabilities. However, it is recommended that the Output Sport team should continue to work on application adjustments or improved versions of the device to enhance validity and reliability further.

## Data Availability

The raw data supporting the conclusions of this article will be made available by the authors, without undue reservation.

## References

[B1] Allen S. King M. Yeadon F. “Tradeoffs between horizontal and vertical velocity during triple jumping,” in Pre-Olympic Congress on Sports Science and Computer Science in Sport (IACSS 2012), UK - United Kingdom, July 2012, 178–180.

[B2] Arampatzis A. Schade F. Walsh M. Bruggemann G. P. (2001). Influence of leg stiffness and its effect on myodynamic jumping performance. J. Electromyogr. Kinesiol. 11 (5), 355–364. 10.1016/s1050-6411(01)00009-8 11595555

[B3] Ball N. B. Zanetti S. (2012). Relationship between reactive strength variables in horizontal and vertical drop jumps. J. Strength Cond. Res. 26 (5), 1407–1412. 10.1519/JSC.0b013e3182510870 22395278

[B4] Balsalobre-Fernandez C. Kuzdub M. Poveda-Ortiz P. del Campo-Vecino J. (2016). Validity and reliability of the PUSH wearable device to measure movement velocity during the back squat exercise. J. Strength Cond. Res. 30 (7), 1968–1974. 10.1519/jsc.0000000000001284 26670993

[B5] Banyard H. G. Nosaka K. Haff G. G. (2017). Reliability and validity of the load-velocity relationship to predict the 1RM back squat. J. Strength Cond. Res. 31 (7), 1897–1904. 10.1519/JSC.0000000000001657 27669192

[B6] Bobbert M. F. Huijing P. A. van Ingen Schenau G. J. (1987). Drop jumping. II. The influence of dropping height on the biomechanics of drop jumping. Med. Sci. Sports Exerc. 19 (4), 339–346. 10.1249/00005768-198708000-00004 3657482

[B7] Bosco C. Luhtanen P. Komi P. V. (1983). A simple method for measurement of mechanical power in jumping. Eur. J. Appl. Physiol. Occup. Physiol. 50 (2), 273–282. 10.1007/BF00422166 6681758

[B8] Buckley C. O'Reilly M. A. Whelan D. Farrell A. V. Clark L. Longo V. “Binary classification of running fatigue using a single inertial measurement unit,” in 2017 Ieee 14th International Conference on Wearable and Implantable Body Sensor Networks (Bsn), Eindhoven, Netherlands, May 2017 (IEEE), 197–201. 10.1109/BSN41205.2017

[B9] Busko K. Madej A. Mastalerz A. (2010). Effects of the cycloergometer exercises on power and jumping ability measured during jumps performed on a dynamometric platform. Biol. Sport 27 (1), 35–40. 10.5604/20831862.907789

[B10] Byrne D. J. Browne D. T. Byrne P. J. Richardson N. (2017). Interday reliability of the reactive strength index and optimal drop height. J. Strength Cond. Res. 31 (3), 721–726. 10.1519/jsc.0000000000001534 27379959

[B11] Choukou M. A. Laffaye G. Taiar R. (2014). Reliability and validity of an accelerometric system for assessing vertical jumping performance. Biol. Sport 31 (1), 55–62. 10.5604/20831862.1086733 24917690 PMC3994586

[B12] Ebben W. P. Petushek E. J. (2010). Using the reactive strength index modified to evaluate plyometric performance. J. Strength Cond. Res. 24 (8), 1983–1987. 10.1519/JSC.0b013e3181e72466 20634740

[B13] Frayne D. H. Zettel J. L. Beach T. A. C. Brown S. H. M. (2021). The influence of countermovements on inter-segmental coordination and mechanical energy transfer during vertical jumping. J. Mot. Behav. 53 (5), 545–557. 10.1080/00222895.2020.1810611 32862794

[B14] Gillen Z. M. Jahn L. E. Shoemaker M. E. McKay B. D. Mendez A. I. Bohannon N. A. (2019). Effects of eccentric preloading on concentric vertical jump performance in youth athletes. J. Appl. Biomech. 35 (5), 327–335. 10.1123/jab.2018-0340 31541066

[B15] Glatthorn J. F. Gouge S. Nussbaumer S. Stauffacher S. Impellizzeri F. M. Maffiuletti N. A. (2011). Validity and reliability of optojump photoelectric cells for estimating vertical jump height. J. Strength Cond. Res. 25 (2), 556–560. 10.1519/jsc.0b013e3181ccb18d 20647944

[B16] Gu C. Y. Li X. R. Lai C. T. Gao J. J. Wang I. L. Wang L. I. (2021). Sex disparity in bilateral asymmetry of impact forces during height-adjusted drop jumps. Int. J. Environ. Res. Public Health 18 (11), 5953. 10.3390/ijerph18115953 34206107 PMC8199539

[B17] Haynes T. Bishop C. Antrobus M. Brazier J. (2019). The validity and reliability of the My Jump 2 app for measuring the reactive strength index and drop jump performance. J. Sports Med. Phys. Fit. 59 (2), 253–258. 10.23736/S0022-4707.18.08195-1 29589412

[B18] Hobara H. Inoue K. Muraoka T. Omuro K. Sakamoto M. Kanosue K. (2010). Leg stiffness adjustment for a range of hopping frequencies in humans. J. Biomech. 43 (3), 506–511. 10.1016/j.jbiomech.2009.09.040 19879582

[B19] Hopkins W. G. (2010). Linear models and effect magnitudes for research, clinical and practical applications. Sports Sci. 14, 49–57.

[B20] Horita T. Komi P. V. Hämäläinen I. Avela J. (2003). Exhausting stretch-shortening cycle (SSC) exercise causes greater impairment in SSC performance than in pure concentric performance. Eur. J. Appl. Physiol. 88 (6), 527–534. 10.1007/s00421-002-0716-z 12560951

[B21] Hughes L. J. Peiffer J. J. Scott B. R. (2019). Retracted: reliability and validity of using the push band v2.0 to measure repetition velocity in free-weight and smith machine exercises. J. Strength Cond. Res. 10.1519/jsc.0000000000003436 31860535

[B22] Jarvis M. M. Graham-Smith P. Comfort P. (2016). A methodological approach to quantifying plyometric intensity. J. Strength Cond. Res. 30 (9), 2522–2532. 10.1519/JSC.0000000000000518 24787677

[B23] Johnston W. O'Reilly M. Argent R. Caulfield B. (2019). Reliability, validity and utility of inertial sensor systems for postural control assessment in sport science and medicine applications: a systematic review. Sports Med. 49 (5), 783–818. 10.1007/s40279-019-01095-9 30903440

[B24] Jorgensen S. L. Bojsen-Moller J. Skalgard T. Olsen H. B. Aagaard P. (2021). Dual vs single force plate analysis of human drop jumping. Transl. Sports Med. 4 (5), 637–645. 10.1002/tsm2.255

[B25] Koo T. K. Li M. Y. (2016). A guideline of selecting and reporting intraclass correlation coefficients for reliability research. J. Chiropr. Med. 15 (2), 155–163. 10.1016/j.jcm.2016.02.012 27330520 PMC4913118

[B26] Lake J. P. Augustus S. Austin K. Mundy P. McMahon J. J. Comfort P. (2018). The validity of the push band 2.0 during vertical jump performance. Sports (Basel) 6 (4), 140. 10.3390/sports6040140 PMC6316333 30400613

[B27] Lake J. Augustus S. Austin K. Comfort P. McMahon J. Mundy P. (2019). The reliability and validity of the bar-mounted PUSH Band(TM) 2.0 during bench press with moderate and heavy loads. J. Sports Sci. 37 (23), 2685–2690. 10.1080/02640414.2019.1656703 31418312

[B28] Li R. T. Kling S. R. Salata M. J. Cupp S. A. Sheehan J. Voos J. E. (2016). Wearable performance devices in sports medicine. Sports Health. 8 (1), 74–78. 10.1177/1941738115616917 26733594 PMC4702159

[B29] Linthorne N. P. (2001). Analysis of standing vertical jumps using a force platform. Am. J. Phys. 69(11), 1198–1204. 10.1119/1.1397460

[B30] Lloyd R. S. Oliver J. L. Hughes M. G. Williams C. A. (2011). Specificity of test selection for the appropriate assessment of different measures of stretch-shortening cycle function in children. J. Sports Med. Phys. Fit. 51 (4), 595–602. 22212261

[B31] Markovic G. Mikulic P. (2010). Neuro-musculoskeletal and performance adaptations to lower-extremity plyometric training. Sports Med. 40 (10), 859–895. 10.2165/11318370-000000000-00000 20836583

[B32] McMahon T. A. Cheng G. C. (1990). The mechanics of running: how does stiffness couple with speed? J. Biomech. 23 (1), 65–78. 10.1016/0021-9290(90)90042-2 2081746

[B33] Montalvo S. Gonzalez M. P. Dietze-Hermosa M. S. Eggleston J. D. Dorgo S. (2021). Common vertical jump and reactive strength index measuring devices: a validity and reliability analysis. J. Strength Cond. Res. 35 (5), 1234–1243. 10.1519/jsc.0000000000003988 33629975

[B34] Montoro-Bombú R. de la Paz Arencibia L. Buzzichelli C. Miranda-Oliveira P. Fernandes O. Santos A. (2022). The validity of the push band 2.0 on the reactive strength index assessment in drop jump. Sensors 22 (13), 4724. 10.3390/s22134724 35808221 PMC9269219

[B35] O'Reilly M. Whelan D. Chanialidis C. Friel N. Delahunt E. Ward T. (2015). “Evaluating squat performance with a single inertial measurement unit,” in 2015 Ieee 12th International Conference on Wearable and Implantable Body Sensor Networks (Bsn), Cambridge, MA, USA, July 2015 (IEEE), 1–25. 10.1109/BSN35574.2015

[B36] O'Reilly M. A. Whelan D. F. Ward T. E. Delahunt E. Caulfield B. (2017a). Technology in strength and conditioning tracking lower-limb exercises with wearable sensors. J. Strength Cond. Res. 31 (6), 1726–1736. 10.1519/JSC.0000000000001852 28538326

[B37] O'Reilly M. A. Whelan D. F. Ward T. E. Delahunt E. Caulfield B. M. (2017b). Classification of deadlift biomechanics with wearable inertial measurement units. J. Biomech. 58, 155–161. 10.1016/j.jbiomech.2017.04.028 28545824

[B38] O'Reilly M. Caulfield B. Ward T. Johnston W. Doherty C. (2018). Wearable inertial sensor systems for lower limb exercise detection and evaluation: a systematic review. Sports Med. 48 (5), 1221–1246. 10.1007/s40279-018-0878-4 29476427

[B39] Orser K. Agar-Newman D. J. Tsai M.-C. Klimstra M. (2020). The validity of the Push Band 2.0 to determine speed and power during progressively loaded squat jumps. Sports Biomech., 1–9. 10.1080/14763141.2020.1829691 33118478

[B40] Pueo B. Penichet-Tomas A. Jimenez-Olmedo J. M. (2020). Reliability and validity of the Chronojump open-source jump mat system. Biol. Sport 37 (3), 255–259. 10.5114/biolsport.2020.95636 32879547 PMC7433325

[B41] Ramirez-Campillo R. Alvarez C. Garcia-Pinillos F. Sanchez-Sanchez J. Yanci J. Castillo D. (2018). Optimal reactive strength index: is it an accurate variable to optimize plyometric training effects on measures of physical fitness in young soccer players? J. Strength Cond. Res. 32 (4), 885–893. 10.1519/jsc.0000000000002467 29389692

[B42] Reimers A. K. Heidenreich V. Bittermann H. J. Knapp G. Reimers C. D. (2021). Accelerometer-measured physical activity and its impact on sleep quality in patients suffering from restless legs syndrome. BMC Neurol. 21 (1), 90. 10.1186/s12883-021-02115-w 33632158 PMC7908727

[B43] Salami S. Wei J. Regan M. Scherr D. Siddiqui J. Kearney M. (2010). Body mass index and prostate size improve performance of a prostate cancer risk calculator at high levels of sensitivity for predicting prostate cancer at initial prostate biopsy: Results from a prospective, multi-center cohort. J. Urol. 183 (4), E818–E819. 10.1016/j.juro.2010.02.2180

[B44] Shrout P. E. Fleiss J. L. (1979). Intraclass correlations: uses in assessing rater reliability. Psychol. Bull. 86 (2), 420–428. 10.1037//0033-2909.86.2.420 18839484

[B45] Turki O. Chaouachi A. Drinkwater E. J. Chtara M. Chamari K. Amri M. (2011). Ten minutes of dynamic stretching is sufficient to potentiate vertical jump performance characteristics. J. Strength Cond. Res. 25 (9), 2453–2463. 10.1519/JSC.0b013e31822a5a79 21792071

[B46] Wallace B. J. Kernozek T. W. White J. M. Kline D. E. Wright G. A. Peng H. T. (2010). Quantification of vertical ground reaction forces of popular bilateral plyometric exercises. J. Strength Cond. Res. 24 (1), 207–212. 10.1519/JSC.0b013e3181c3b841 19924006

[B47] Wang I. L. Chen Y. M. Zhang K. K. Li Y. G. Su Y. Wu C. (2021). Influences of different drop height training on lower extremity kinematics and stiffness during repetitive drop jump. Appl. Bionics Biomech. 2021, 1–9. 10.1155/2021/5551199 PMC7952188 33747121

[B48] Whelan D. O'Reilly M. Huang B. Q. Giggins O. Kechadi T. Caulfield B. “Leveraging IMU data for accurate exercise performance classification and musculoskeletal injury risk screening,” in 2016 38th Annual International Conference of the Ieee Engineering in Medicine and Biology Society (Embc), Orlando, FL, USA, August 2016 (IEEE), 659–662. 10.1109/EMBC.2016.7590788 28268414

